# Supplementary material for: Global Analysis of RNA-Dependent RNA Polymerase-Dependent Small RNAs Reveals New Substrates and Functions for These Proteins and SGS3 in Arabidopsis
Source: Noncoding RNA. 2021 Apr 27;7(2):28. doi: 10.3390/ncrna7020028 (PMC8167712; doi:10.3390/ncrna7020028)
Supplement: Supplementary file 1 [file ncrna-07-00028-s001.zip › Supplement-PUB/Supplement Figure legends (with figures).pdf]

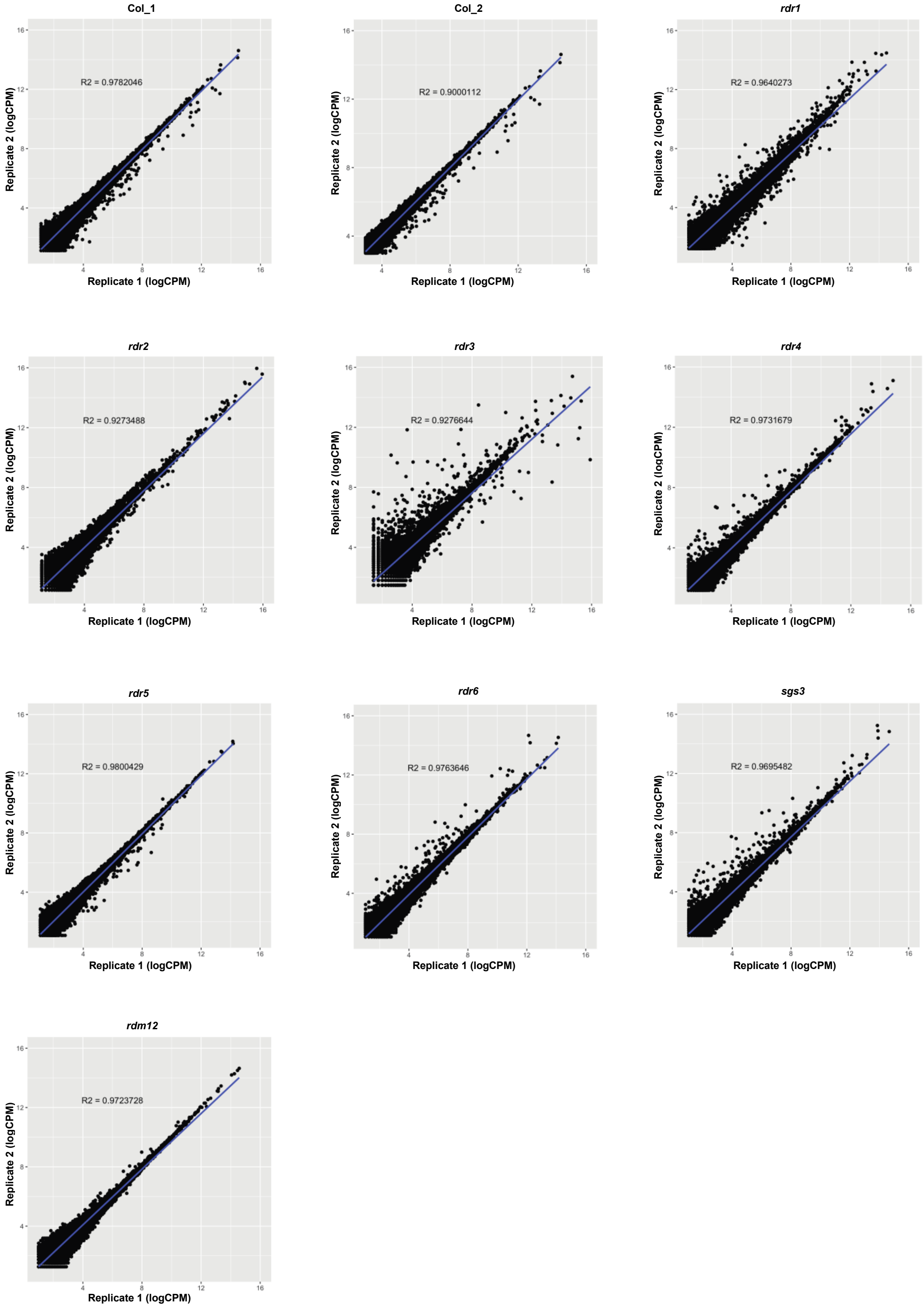

Figure S1. Correlation analysis of smRNA-seq libraries. X-axis and y-axis indicate logCPM (Counts Per Million mapped reads) of smRNA-seq libraries using the R package edgeR. Genotypes for the smRNA-seq libraries are shown above each scatter plot, Col_1 are Col-0 samples prepared with *rdr1, 2, 4 – 6, sgs3, and rdm12*, Col_2 are Col-0 samples prepared with *rdr3* mutants. Coefficient of correlation (R^2) are shown on each plot.

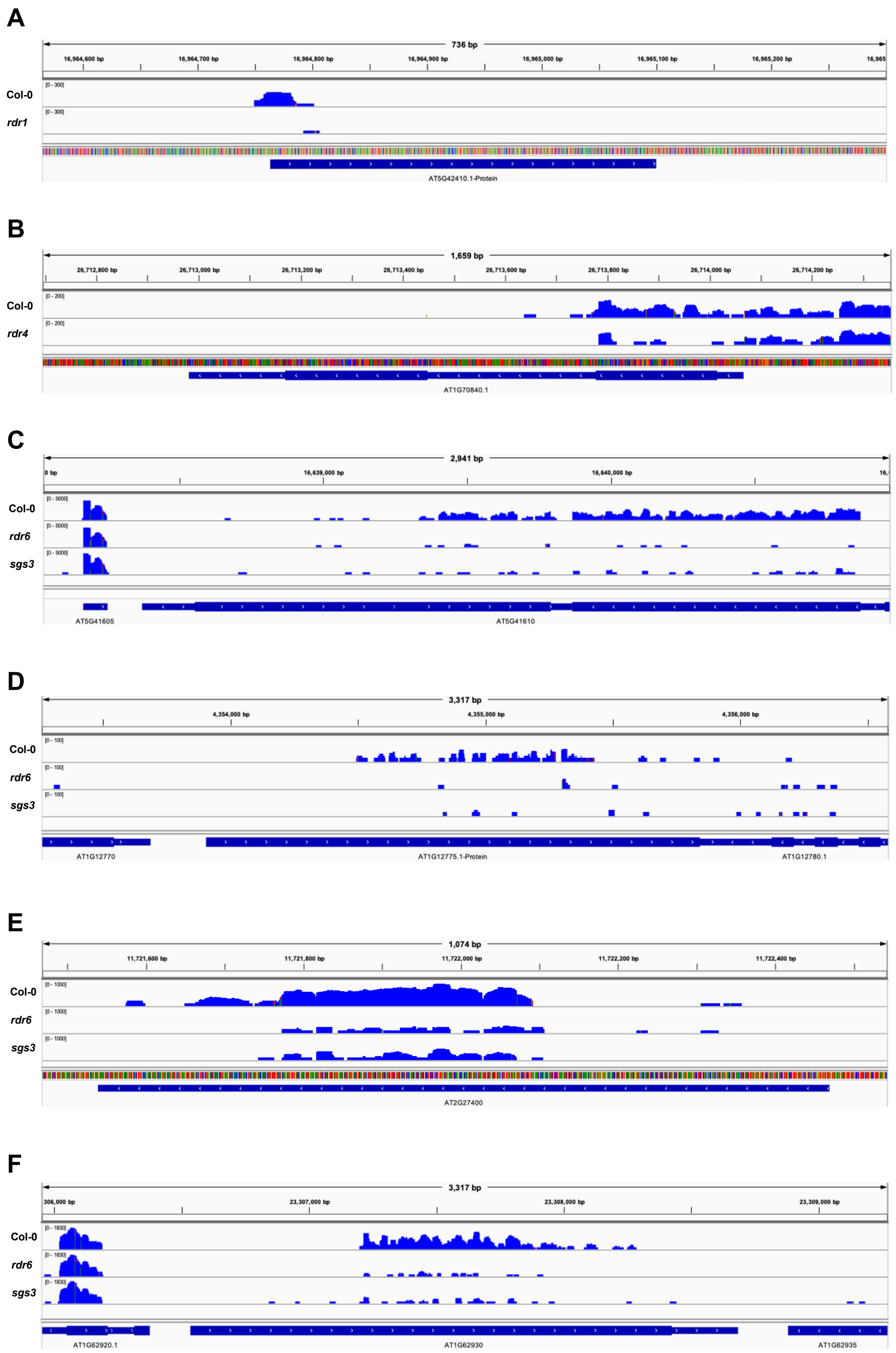

Figure S2. Browser views at example transcripts demonstrating loss of smRNA enrichment in mutants compared to Col-0. Each mutant genotype is indicated on the left of each browser window. Example transcripts are **(A)** *AT5G42410*, **(B)** *AT1G70840*, **(C)** Antisense of a gene *AT5G41610*, **(D)** AT1G12775, **(E)** AT2G27400, and **(F)** AT1G62930.

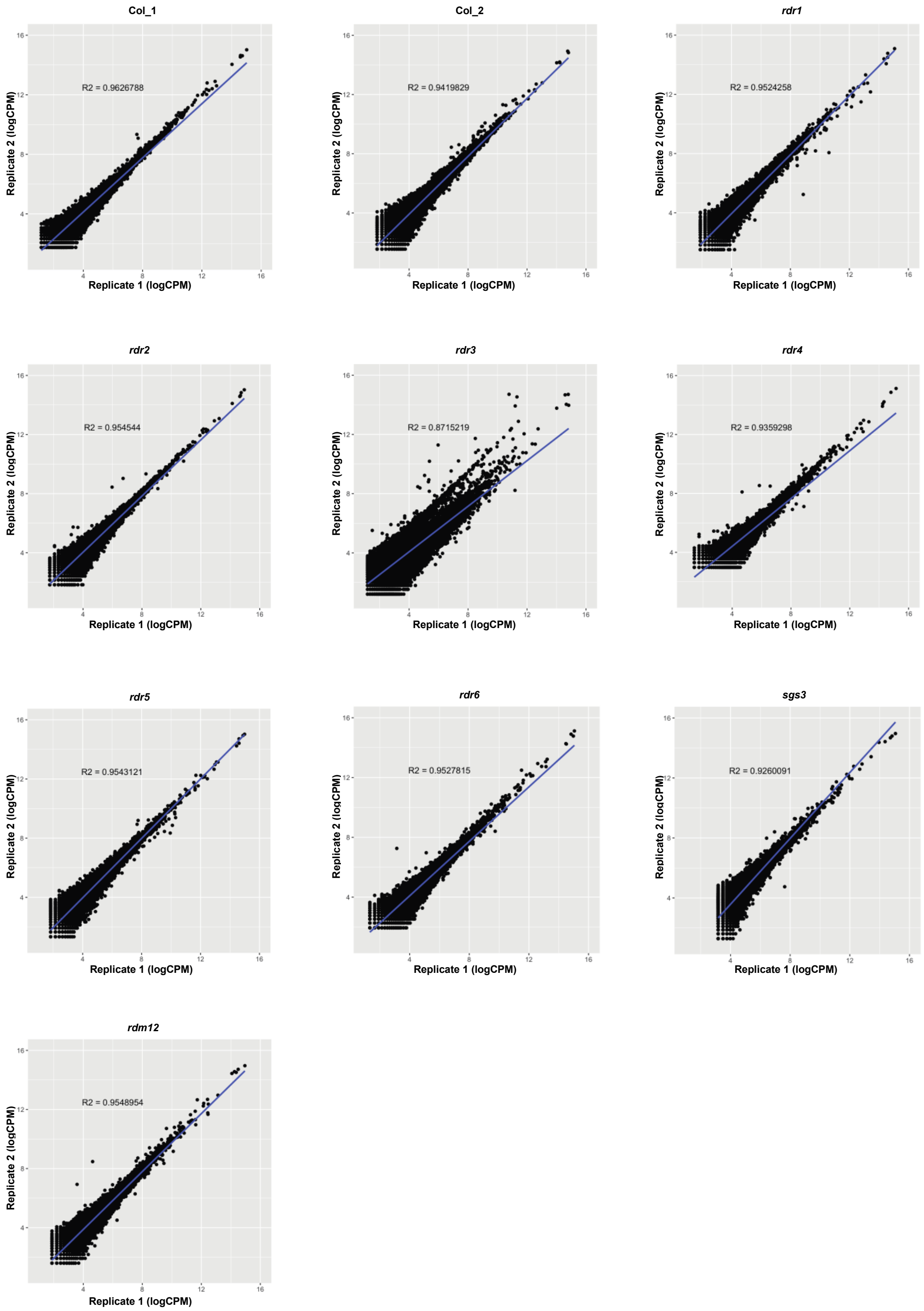

Figure S3. Correlation analysis of total RNA-seq libraries. X-axis and y-axis indicate logCPM (Counts Per Million mapped reads) of total RNA-seq libraries using the R package edgeR. Genotypes for the total RNA-seq libraries are shown above each scatter plot, Col_1 are Col-0 samples prepared with *rdr1, 2, 4 – 6, sgs3, and rdm12*, Col_2 are Col-0 samples prepared with *rdr3* mutants. Coefficient of correlation (R^2) are shown on each plot.
